# Evaluation of the Canal Transportation Following Glide Path Preparation with Different Rotary Systems: A Comparative Study

**DOI:** 10.1155/2022/8087378

**Published:** 2022-06-30

**Authors:** Mohsen Aminsobhani, Fatemeh Hamidzadeh, Arvin Rezaei Avval, Farid Merrikhi, Ehsan Sadri

**Affiliations:** ^1^Faculty of Dentistry, Dental Research Center, AJA and Tehran University of Medical Sciences, Tehran, Iran; ^2^Department of Endodontics, Faculty of Dentistry, Tehran University of Medical Science, Tehran, Iran; ^3^Faculty of Dentistry, AJA University of Medical Sciences, Tehran, Iran; ^4^Department of Physics, Faculty of Science, Central Tehran Branch of Azad University, Tehran, Iran

## Abstract

**Objectives:**

Ideal root canal shaping might be more challenging particularly in cases with severely curved canals or complex anatomical variations. Glide path preparation is suggested as a critical step to achieve ideal canal preparation. The present study is aimed at evaluating transportation at different levels of the canal following glide path preparation by five different path finders.

**Materials and Methods:**

The study was conducted on 100 S-shaped canal simulator blocks. Glide path was prepared in five groups including (1) Scout RaCe (^#^10 and ^#^15, 0.02), (2) One G (^#^14, 0.03), (3) PathFile (^#^13 and ^#^16, 0.02), (4) GPS (^#^15, 0.03), and (5) K file (^#^15, 0.02) (Control group). The first four groups were NiTi rotary instruments, while the last group was a stainless steel hand file. The aforementioned files were used after canal negotiation by a ^#^10 stainless steel hand file. Before- and after-preparation photos were taken and were superimposed in Adobe PhotoShop CC 2019. Transportation measurements were conducted in Digimizer. Absolute canal transportation was calculated at 10 cross-sections. Intergroup and intragroup data analysis were conducted using one-way and repeated measures ANOVA tests, respectively, in SPSS 26.0. The significance level was set to 0.05.

**Results:**

Although K file led to significantly more transportation in the apical and middle thirds (*p* < 0.001), rotary groups were not statistically different. In the coronal third, K files led to significantly more transportation compared to Scout RaCe and PathFile (*p* < 0.05).

**Conclusion:**

Within the limitations of the present study, regardless of the recruited rotary system, glide path preparation using NiTi rotary instruments leads to less canal transportation compared to stainless steel hand files.

## 1. Introduction

An ideal root canal preparation is essential in order to facilitate irrigation, disinfection, and proper obturation of the canal, thus preventing or eliminating apical periodontitis [1]. Meanwhile, respecting the original canal anatomy, the position, and size of the apical foramen are mandatory to achieve an ideal preparation [2]. Achieving an ideal root canal preparation might be challenging in severely curved or double-curved canals [3]. Considering the quite high prevalence of double-curved or S-curved canal morphology [4], management of such cases still remains a great challenge to endodontists.

A glide path is defined as a smooth radicular tunnel extending from the canal orifice to the physiologic terminus [5]. Creating a glide path prior to the process of cleaning and shaping by NiTi rotary instruments has been shown to reduce torsional stress and can increase the life span of a rotary instrument up to 6 times [6]. Maintaining the original canal curvature means less canal transportation, less ledge formation, and less root perforations occur when an effective glide path is prepared before any further instrumentation [7]. An established glide path allows for predictable radicular cleaning and shaping and is strongly suggested to be the starting point of all root canal preparations.

Glide path creation can be carried out with either precurved stainless steel K files or NiTi rotary path finder instruments [5]. K files have been recommended by several authors [6, 8, 9], whereas nickel-titanium (NiTi) rotary instruments have been shown to be faster and lead to fewer procedural accidents, particularly in severely curved cases, as a result of the higher flexibility of the alloy [10–12]. Glide path preparation using stainless steel hand files may have some advantages, including improved tactile sensation, appreciation of anatomic curvatures, decreased risk of file fracture, negotiation of canal blockages, and decreased cost. The disadvantages include operator and hand fatigue; the risk of canal aberrations with the use of larger file sizes; and significant changes in the original canal anatomy, combined with the increased apical extrusion of debris [12, 13].

Studies have shown that glide path creation with NiTi instruments is faster and causes fewer procedural errors than K files [13]. It has been suggested that PathFile (PFs; Dentsply Sirona, York, PA) can prepare a glide path with fewer irregularities and better conservation of the original canal anatomy [14]. One G, PathFile, Scout RaCe, and GPS are among the pathfinder rotary instruments mostly used by clinicians.

One G (Micro-Mega, Besançon Cedex, France) is a 0.03 tapered NiTi glide path file with a tip size of 0.14 mm. It is suggested to be used with a torque limit of 1.2 Ncm and a speed of 250–400 rpm. PathFile (PF) (Dentsply Maillefer, Ballaigues, Switzerland) NiTi rotary instruments are used for creating an initial glide path mechanically. The system consists of 3 path finder instruments with ISO 13, 16, and 19 tip sizes, a 0.02 taper, and a square cross section. PathFile is suggested to be used with a torque limit of 5 and 6 Ncm and a speed of 300 rpm. Scout RaCe (FKG Dentaire, La Chaux-de-Fonds, Switzerland) rotary pathfinding instruments are 3 instruments, with ISO 10, 15, and 20 tip sizes and a 0.02 constant taper, manufactured from conventional NiTi. Scout RaCe is suggested to be used with a torque limit of 1.5 Ncm and a speed of 800 rpm. PathFile and Scout RaCe instruments have 4 cutting edges with a square cross section. The GPS files (NEOLIX, Châtres-la-Forêt, France) are NiTi rotary path files with a tip size of 0.15 mm and a 0.03 constant taper. It is used with continuous rotation, a torque limit of 1.5 Ncm, and a speed of 300–500 rpm.

K-files (Mani K-files, Mani, Japan) are hand files manufactured from twisted square stainless steel blanks. They are the standard instruments used for root canal preparation. Because of the sharp and mirror-like finished edges with high ductility, these instruments have excellent working characteristics. K files are used with a watch-winding hand motion and can also be reciprocated.

There are a couple of studies investigating the deviation of the canal during glide path preparation [10, 15, 16], most of which are aimed at comparing a specific rotary system with hand instruments. However, there is not enough evidence available to determine whether a specific rotary system outperforms the others in terms of canal transportation. Thus the present study focused on a comparison between 5 different path finders (Scout RaCe, One G, Path File, GPS, and MA NI K file) in terms of canal transportation at different canal levels to investigate if there is any difference among the aforementioned systems. The null hypothesis would be that there is no difference among different pathfinders in terms of canal transportation.

## 2. Materials and Methods

**Figure 1:**
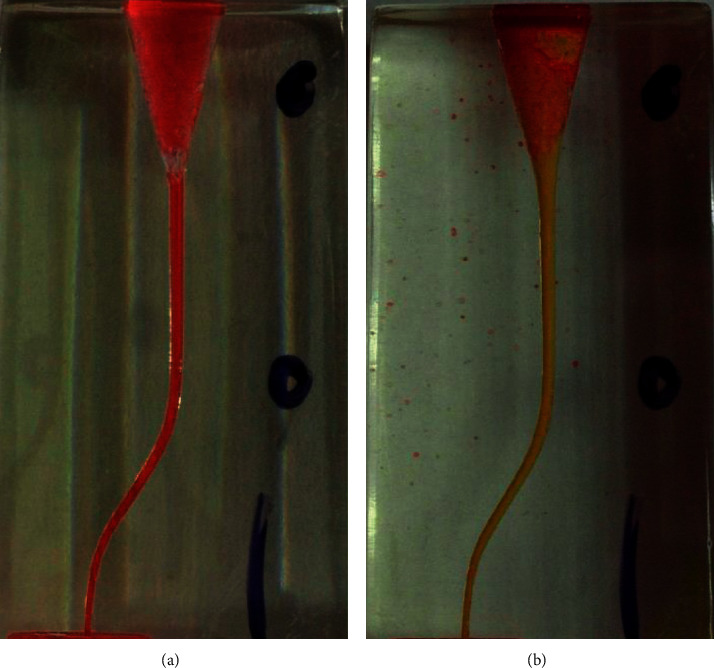
Before- (a) and after- (b) preparation photos of one of the blocks after painting with dyes; blocks were painted with red dye before instrumentation while they were painted with yellow dye after glide path creation.

**Figure 2:**
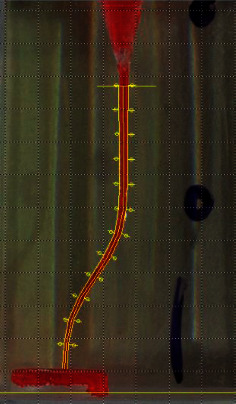
The pattern used to measure transportation at each cross-section. From the apical part of simulated canal: the first, the second, and the third cross sections were assumed as the apical third; the forth, the fifth, the sixth, and the seventh cross sections were assumed as the middle third; the eighth, the ninth, and the tenth were assumed as the coronal third.

The present study was conducted on 100 S-shaped canal simulator blocks (E-block, Acadental, USA), assigned to 5 groups (20 blocks were assigned to each group): (1) Scout RaCe (^#^10 and ^#^15, 0.02), (2) One G (^#^14, 0.03), (3) PathFile (^#^13 and ^#^16, 0.02), (4) GPS (^#^15, 0.03), (5) K file (^#^15, 0.02) (Control). The first four groups were NiTi rotary instruments, while the last group was a stainless steel hand file. Canal simulator endo-blocks were used in the present study to standardize the methodology and control confounding variables [17, 18]. In each group, the canal was negotiated by a ^#^10 stainless steel K-file (MANI K-files, MANI, Japan) and then rotary instruments were introduced into the canal with gentle strokes till the working length was reached. Glide path preparation was carried out in the first group by Scout RaCe ^#^10 and ^#^15; in the second group by One G file (^#^14, 0.03) as a single file; in the third group by PathFile ^#^13 and ^#^16; in the fourth group by GPS (^#^15, 0.03); and in the fifth group by ^#^15 K-file (MANI K-files, MANI, Japan). K-files were introduced into the canal with watch-winding motion and circumferential motion, subsequently. Rotary files were used in order to prepare a glide path according to the manufacturer's catalogue (Table 1), using the Endo Pilot motor (Schlumbohm, Brokstedt, Germany). The canal of blocks was painted with red dye and a photo was taken from the block before preparation. Once glide path preparation was performed the canal was painted with yellow dye and another photo was taken (Figure 1). These photos were taken by the Dino-Lite AM4113TL stereomicroscope (AnMo Electronics Corporation, New Taipei City, Taiwan) in a reproducible condition. Before- and after-preparation pictures were superimposed in Adobe PhotoShop CC 2019 (Adobe Inc., San Jose, California). Blocks were assessed at ten cross-sections with one mm intervals in Digimizer image analysis software (MedCalc Software Ltd.). Absolute canal transportation at each cross-section was calculated as the half of the absolute value of the difference between left and right-side transportation at that cross-section. The mean of the absolute canal transportation at the first, second, and third cross-sections was assumed as apical canal transportation. The mean of the absolute canal transportation at the fourth, fifth, sixth, and seventh cross-sections was assumed as middle canal transportation. The mean of the absolute canal transportation at the eighth, ninth, and tenth cross-sections was assumed as coronal canal transportation (Figure 2). Data analysis was conducted in the SPSS 26.0 software (IBM Corporation, New York, United States of America). Intergroup comparison was performed using one-way ANOVA combined with a post hoc Tukey test. Intragroup analysis was conducted using the repeated measures ANOVA test. Statistical significance was set to 0.05.

## 3. Results

**Figure 3:**
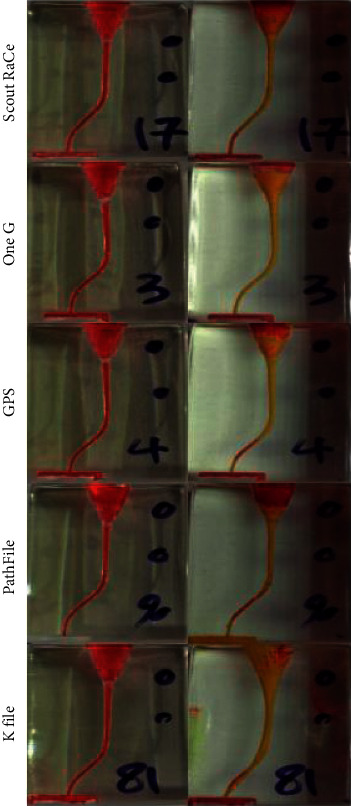
Representative images of before- and after-instrumentation pictures of each experimental group.

A summary of the results in each group (described as Mean ± SD) is presented in Table 2. Representative images of before- and after-instrumentation pictures of each experimental group are illustrated in Figure 3. The minimum mean apical, middle, and coronal transportation values were 0.034 mm, 0.045 mm, and 0.034 mm, respectively, observed in Scout RaCe group. The maximum apical, middle, and coronal mean transportation values were 0.226 mm, 0.236 mm, and 0.098 mm, respectively, observed in K file group (Control).

NiTi path finder files mean transportation at each level was observed as follows: Apical third, PathFile > One *G* > GPS > Scout RaCe, Middle third: GPS > One G > Pathfile > Scout RaCe, Coronal third, One G > GPS > PathFile > Scout RaCe.

### 3.1. Intergroup Analysis

In the apical and middle thirds of the canal, all of the path finders showed significantly less canal transportation compared to the control group (K file) (*p* < 0.001). Pairwise comparisons between NiTi groups did not show any significant differences among them in the apical and middle thirds of the canal. In the coronal third, Scout RaCe, and PathFile were associated with less canal transportation when compared to the control group (*p* < 0.001 and *p* < 0.001, respectively). However, GPS and One G were not significantly different from the control group regarding coronal transportation (*p*=0.088 and *p*=0.354, respectively). Pairwise comparisons between NiTi groups did not show any significant differences among them in the coronal third of the canal.

### 3.2. Intragroup analysis

Intragroup comparisons revealed that canal deviation caused by Scout RaCe and One G was not significantly different throughout the canal; i.e., the apical, middle, and coronal transportation values in each group were not statistically different (*p* > 0.05) (Table 3). GPS showed significantly more transportation in the middle third compared to the apical third of the canal (*p*=0.001). PathFile deviated the coronal third of the canal significantly less than the apical (*p*=0.008) and the middle (*p* < 0.001) thirds of the canal. K file also showed the same pattern as the PathFile (*p* < 0.001).

## 4. Discussion

The present study has focused on the transportation caused by five path finder files, including One G, PathFile, GPS, Scout RaCe, and K file, on different levels of an S-shaped canal.

The major determinants of canal transportation mentioned by previous authors are canal anatomy, instrument design, instrument alloy, and instrumentation technique [19]. On the other hand, as canal modifications during glide path preparation are expected to be accentuated during canal preparation with larger instruments, all efforts must be made to minimize canal transportation during glide path preparation. In the present study the S-shaped, double-curved, canal simulator endo-blocks were recruited, which is known as a challenge to endodontists. Study results suggest the fact that all of the NiTi files used in the experimental groups respected the canal anatomy at different levels of the curvature. However, K files transported the canal at different levels significantly more than the other experimental groups.

As apical transportation may compromise the apical seal of the obturation, it might be of great concern in terms of treatment outcome [20]. Among the NiTi rotary files, Scout RaCe had the least, although not statistically significant, mean apical transportation (0.034 mm). The next group was GPS with 0.045 mm followed by One G with 0.050 mm and PathFile with 0.058 mm mean apical transportation. The data suggests that Scout RaCe may respect the apical anatomy more than the other groups. However the other NiTi pathfinders can be used to prepare a glide path with the least risk of significant apical transportation. The mean apical transportation in the K file group was 0.226 mm where the diameter of the cross section of the canal at baseline was 0.200 mm. It means that, on average, K file has doubled the diameter of the canal during glide path preparation. Considering the primary objectives of glide path preparation, apical transportation as observed in K file group would not be favorable. In a previous study, Zheng et al. [7] reported that PathFile led to less canal transportation in comparison with K file, which is in agreement with the present study's results. These findings are mainly attributed to the high flexibility of NiTi instruments.

Considering the main objectives of cleaning and shaping by Schilder [21] and the specific design of the studied instruments, in the case of an ideal preparation, the clinician may not expect the apical transportation to be more than or even equal to coronal transportation. The data suggests that in K file and PathFile groups, apical transportation was more (2.3 times and 1.5 times, respectively) than coronal transportation. The higher tendency of PathFile to deviate from the original shape of the canal might be attributed to the short transitional angle which may act as an active tip. This is in agreement with a previous study's findings, which had compared PathFile with Scout RaCe and reported that PathFile is more aggressive than Scout RaCe [15]. Coronal, middle, and apical transportation were not statistically different in the Scout RaCe and One G group. This means that even the alternating curvature of the canal did not affect the transportation values. This might be due to the high flexibility of Scout RaCe which has been mentioned previously in several studies [15, 16, 22]. Except for the One G group, all of the groups had the highest mean transportation at the middle third. This is mainly due to the morphological characteristics of the studied S-shaped canal; i.e., the zone of alternating curvature of the canal is the middle third. The extremely high middle-third transportation observed in the K file group means that K file considerably modifies the original morphology of the curvatures.

The present study has recruited endo-blocks as S-shaped canal simulators. Thus the results must be interpreted with caution. It is suggested for future studies to investigate canal transportation values associated with different path-finding instruments on *ex vivo* models. Further *in vivo* studies are also required to investigate the clinical significance of the observed differences among the path finding instruments; i.e., whether the difference among the path finding instruments in terms of canal transportation affects treatment outcomes significantly.

## 5. Conclusion

Within the limitations of the present study, glide path preparation using K files results in significantly more transportation at different levels of the canal; while none of the NiTi rotary path finding instruments investigated in the present study, including One G, Scout RaCe, GPS, and PathFile, outperformed the others with regard to canal transportation.

## Figures and Tables

**Table 1:** Main characteristics of path finding instruments used in the present study.

Path finding instrument	Manufacturer	Tip size/taper	Speed (rpm)/Torque (Ncm)	Alloy
K file	MANI	^#^10, ^#^15/0.02	—	Stainless steel
Scout RaCe	FKG dentaire	^#^10, ^#^15/0.02	800/1.5	NiTi
PathFile	Dentsply maillefer	^#^13, ^#^16/0.02	300/6	NiTi
One G	Micro mega	^#^14/0.03	400/1.2	NiTi
GPS	NEOLIX	^#^15/0.03	500/1.5	NiTi

**Table 2:** Mean transportation values at apical, middle, and coronal canal thirds for each experimental group.

Group	Apical third transportation (mm)	Middle third transportation (mm)	Coronal third transportation (mm)
Scout RaCe (*n* = 20)	0.034 ± 0.027^a^	0.045 ± 0.024^a^	0.034 ± 0.021^a^
One G (*n* = 20)	0.050 ± 0.029^a^	0.063 ± 0.027^a^	0.069 ± 0.039^ab^
GPS (*n* = 20)	0.045 ± 0.022^a^	0.071 ± 0.035^a^	0.058 ± 0.043^ab^
PathFile (*n* = 20)	0.058 ± 0.025^a^	0.059 ± 0.036^a^	0.037 ± 0.029^a^
K File (*n* = 20) (control)	0.226 ± 0.068^b^	0.236 ± 0.061^b^	0.098 ± 0.074^b^

^*∗*^Different superscript letters on the same column indicate statistical significance. (*p* < 0.05).

**Table 3:** *P*values for each pairwise comparison in the intragroup analysis.

Group	Pairwise comparisons		
	Apical vs. Middle Third Transportation	Apical vs. Coronal Third Transportation	Coronal vs. Middle third transportation
Scout RaCe	0.234	1	0.240
One G	0.132	0.121	1
GPS	0.001^*∗*^	0.562	0.349
PathFile	1	0.008^*∗*^	<0.001^*∗*^
K File	1	<0.001^*∗*^	<0.001^*∗*^

## Data Availability

The data supporting the findings of the present study are available from corresponding author upon request.

## References

[1] Young G. R., Parashos P., Messer H. H. (2007). The principles of techniques for cleaning root canals. *Australian Dental Journal*.

[2] Waplington M., McRobert A. S. (2014). Shaping the root canal system. *British Dental Journal*.

[3] Schäfer E., Vlassis M. (2004). Comparative investigation of two rotary nickel-titanium instruments: protaper versus RaCe. Part 2. cleaning effectiveness and shaping ability in severely curved root canals of extracted teeth. *International Endodontic Journal*.

[4] Willershausen B., Tekyatan H., Kasaj A., Marroquín B. B. (2006). Roentgenographic in vitro investigation of frequency and location of curvatures in human maxillary premolars. *Journal of Endodontics*.

[5] West J. D. (2010). The endodontic glidepath: “secret to rotary safety”. *Dentistry Today*.

[6] Berutti E., Negro A. R., Lendini M., Pasqualini D. (2004). Influence of manual preflaring and torque on the failure rate of protaper rotary instruments. *Journal of Endodontics*.

[7] Zheng L., Ji X., Li C., Zuo L., Wei X. (2018). Comparison of glide paths created with K-files, pathfiles, and the proglider file, and their effects on subsequent waveone preparation in curved canals. *BioMed Central Oral Health*.

[8] Mounce R. (2005). Endodontic K-files: invaluable endangered species or ready for the Smithsonian?. *Dentistry Today*.

[9] Patiño P. V., Biedma B. M., Liébana C. R., Cantatore G., Bahillo J. G. (2005). The influence of a manual glide path on the separation rate of NiTi rotary instruments. *Journal of Endodontics*.

[10] Berutti E., Cantatore G., Castellucci A. (2009). Use of nickel-titanium rotary pathfile to create the glide path: comparison with manual preflaring in simulated root canals. *Journal of Endodontics*.

[11] Jonker C., Van der Vyver P., De Wet F. (2014). The influence of glide path preparation on the failure rate of waveone reciprocating instruments. *SADJ: Journal of The South African Dental Association*.

[12] van der Vyver P., Paleker F., Jonker C. (2015). Comparison of preparation times of three different rotary glide path instrument systems. *South African Dental Journal*.

[13] Paleker F., van der Vyver P. J. (2016). Comparison of canal transportation and centering ability of K-files, proglider file, and G-files: a micro-computed tomography study of curved root canals. *Journal of Endodontics*.

[14] Cantatore G. B. E., Castellucci A. (2010). The pathfiles: a new series of rotary nickel titanium instruments for mechanical pre-flaring and creating the glide path. *Oral Health*.

[15] Ajuz N. C., Armada L., Gonçalves L. S., Debelian G., Siqueira J. F. (2013). Glide path preparation in S-shaped canals with rotary pathfinding nickel-titanium instruments. *Journal of Endodontics*.

[16] Lopes H. P., Elias C. N., Siqueira J. F. (2012). Mechanical behavior of pathfinding endodontic instruments. *Journal of Endodontics*.

[17] Calberson F. L., Deroose C. A., Hommez G. M., Raes H., De Moor R. J. (2002). Shaping ability of GTTM rotary Files in simulated resin root canals. *International Endodontic Journal*.

[18] Yang G. B., Zhou X. D., Zhang H., Wu H. K. (2006). Shaping ability of progressive versus constant taper instruments in simulated root canals. *International Endodontic Journal*.

[19] Bürklein S., Benten S., Schäfer E. (2013). Shaping ability of different single-file systems in severely curved root canals of extracted teeth. *International Endodontic Journal*.

[20] Wu M. K., Fan B., Wesselink P. R. (2000). Leakage along apical root fillings in curved root canals. part I: effects of apical transportation on seal of root fillings. *Journal of Endodontics*.

[21] Schilder H. (1974). Cleaning and shaping the root canal. *Dental Clinics of North America*.

[22] Lopes H. P., Elias C. N., Mangelli M. (2012). Buckling resistance of pathfinding endodontic instruments. *Journal of Endodontics*.

